# Defining female gluteal geometry for preoperative planning in body contouring

**DOI:** 10.1016/j.jpra.2026.05.020

**Published:** 2026-05-13

**Authors:** Edoardo Raposio, Elisa Bertulla

**Affiliations:** a Plastic Surgery Chair, Department of Surgical Sciences and Integrated Diagnostics (DISC), University of Genova, Italy; b Plastic and Reconstructive Surgery Division, IRCCS Azienda Ospedaliera Metropolitana, Genova, Italy

**Keywords:** Buttocks aesthetics, Female beauty, Female profile, Gluteoplasty, Brazilian butt lift

## Abstract

**Introduction:**

The increasing prevalence of procedures aimed at modifying the female body silhouette—particularly gluteoplasty, liposuction, and the Brazilian butt lift (BBL)—highlights the need for a sufficient number of reference points and lines to objectively describe these interventions and assess their outcomes.

**Material and methods:**

For the lateral profile, we identified five horizontal reference lines, all parallel to one another, drawn through what we consider the principal landmarks of the posterior trunk curvature. For the posterior view, five horizontal lines were similarly drawn, parallel to one another, corresponding to key posterior landmarks. After tracing these lines in both the lateral and posterior views, we assessed whether recognizable geometric shapes and/or relationships could be identified among them.

**Results:**

We observed that attractive proportions of the lateral female pelvis can be inscribed within a cone-shaped ovoid oriented at approximately 50° relative to the vertical axis. In the posterior view, rectangles and two ovoids may also be identified.

**Conclusions:**

In our view, describing geometric parameters may contribute to a more systematic and standardized framework for characterization and preoperative planning.

Combining lateral and posterior reference lines allowed identification of multiple geometric configurations, including rectangles and ovoids, which may facilitate visualization of spatial relationships among gluteal structures.

## Introduction

In recent years, we have conducted several studies aimed at identifying geometric forms to which aspects of human morphology can be reduced, with a particular focus on female gluteal shape and proportions considered aesthetically pleasing.^1^, ^2^, ^3^, ^4^, ^5^ In the present study, we sought to further develop these concepts by identifying and describing reference lines and geometric figures that may represent attractive female gluteal morphology. We propose that incorporating these geometric parameters may contribute to a more systematic and standardized classification of the female gluteal region for surgical planning.

## Materials and methods

For the lateral profile, we identified three horizontal reference lines, all parallel to one another, drawn through what we consider the principal landmarks of the posterior trunk curvature. Line A was placed at the upper limit of dorsal concavity (corresponding to the apex of the mammary cone); line II at the apex (deepest point) of dorsal concavity; and line V at the inferior end of the gluteal curvature.

For the posterior view, five horizontal lines were similarly drawn, parallel to one another, corresponding to key posterior landmarks. Line W was placed at waist level (point of greatest concavity); line IC at the apex of the iliac crests; line IS at the level of the anterior superior iliac spines; line GT at the level of the greater trochanters; and line IG tangent to the inferior margin of the infragluteal fold.

After tracing these lines in both the lateral and posterior views, we assessed whether recognizable geometric shapes and/or relationships could be identified among them.

## Results

**Fig. 1:**
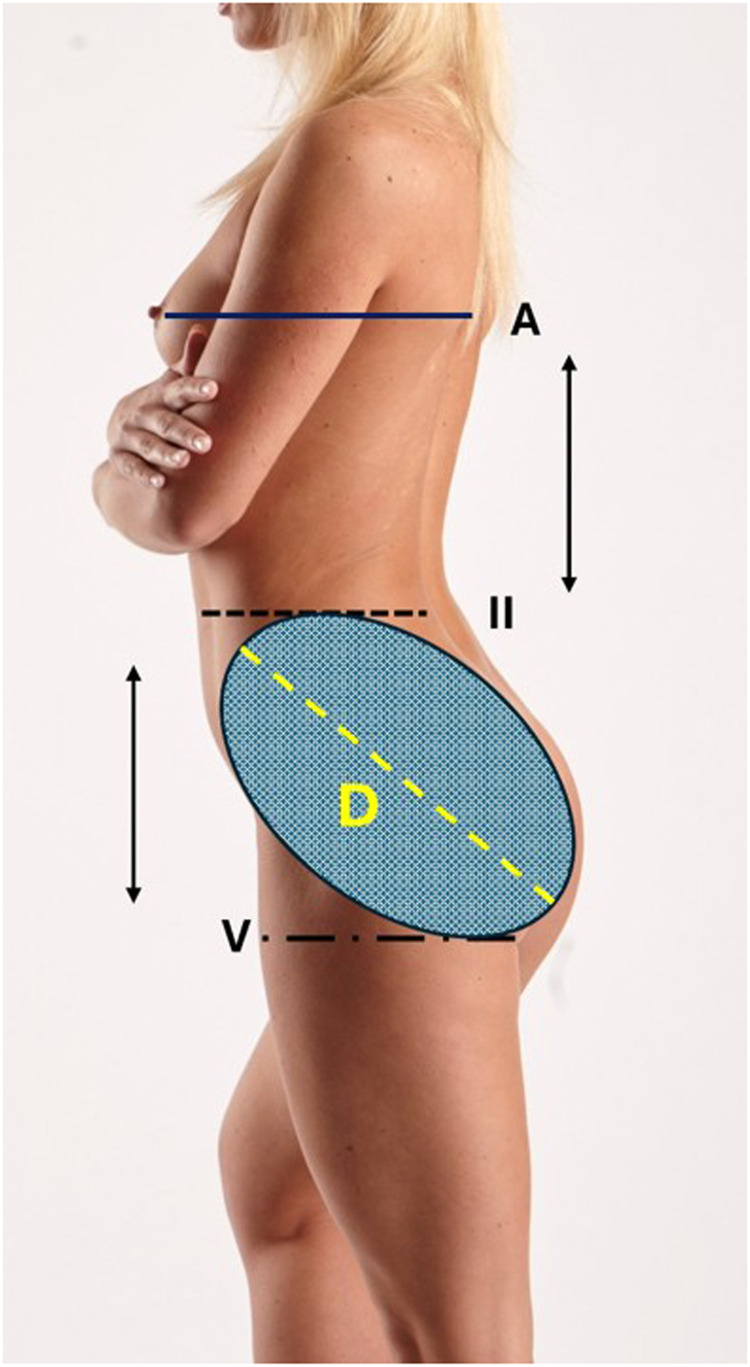
Three horizontal reference lines outlining landmarks of the female trunk in lateral profile: (A) from the onset of dorsal concavity to the apex of breast prominence; (II) from the umbilicus to the point of greatest dorsal concavity; and (V) from the inferior end of gluteal convexity to the point of maximal lateral thigh projection. Within the rectangle bounded by lines II and V, encompassing the entire gluteal curve, a regular oval can be inscribed. Its major axis (D), oriented toward the umbilicus at an angle of approximately 50°, coincides with the rectangle diagonal. The distance between lines II and V equals the distance between the umbilicus and the apex of breast prominence (line A).

In the female lateral profile, five essentially horizontal and parallel reference lines can be identified. These lines pass through prominent points along the anterior and posterior contours. Within the rectangle defined by lines II and V—encompassing the entire gluteal curvature—a regular oval can be inscribed, with its superior and inferior apices corresponding to the lateral abdominal and gluteal curves (Fig. 1). The major axis of this oval coincides with the rectangle diagonal and is oriented toward the umbilicus at approximately 50° relative to the vertical axis. This cone-shaped geometric model accounts for two aesthetic features of the female lateral profile:^1^ the point of maximal lateral buttock projection does not typically lie on a line perpendicular to the vertical axis but is located slightly inferior to it, consistent with the inclination of the ovoid; and^2^ the anterior abdominal profile is often perceived as more aesthetically pleasing when slightly convex—resembling the ovoid apex—rather than straight, which is more commonly associated with masculine contours.

**Fig. 2:**
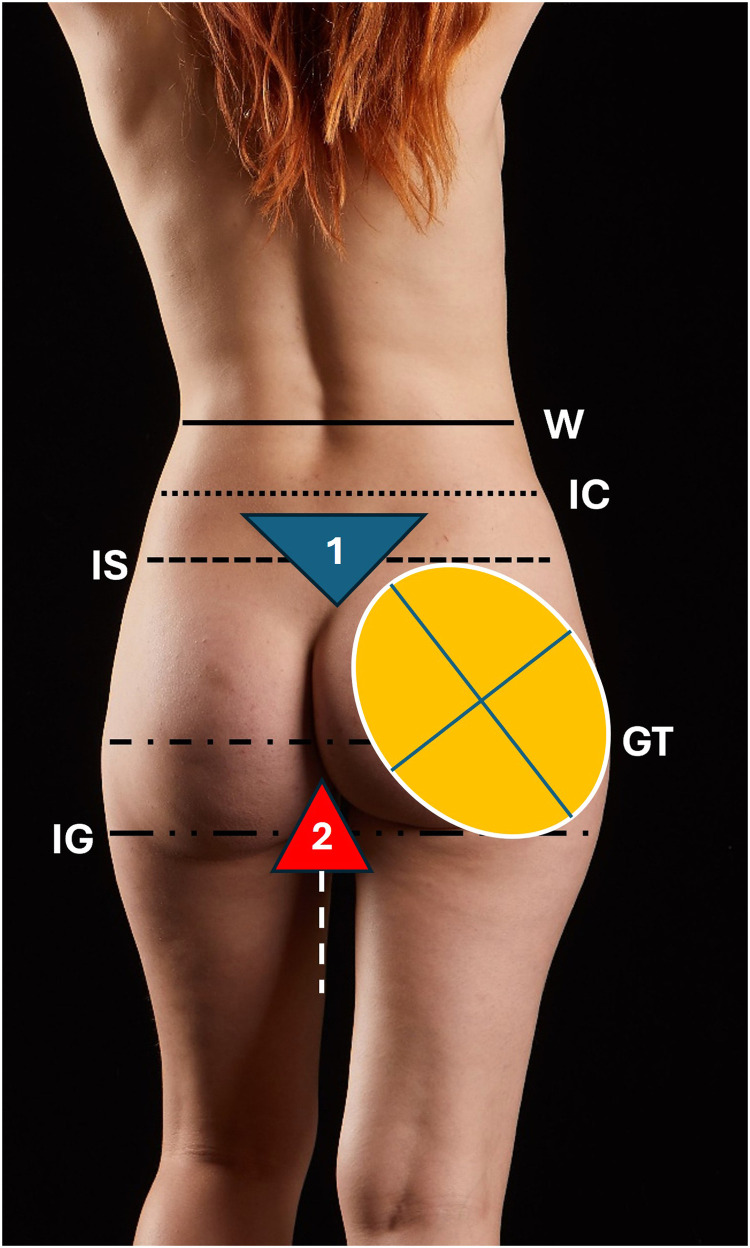
Principal reference lines of the posterior gluteal region: W (waist), IC (iliac crest apex), IS (anterior superior iliac spine), GT (greater trochanter), and IG (inferior margin of the infragluteal fold). The distance between W and IC equals that between IC and IS. Overall gluteal convexity extends from W to IG, whereas the true gluteal region spans from IS to IG. Triangle 1 is defined by the sacral depression, with the lateral vertices corresponding to the bilateral dimples of Venus and the inferior vertex at the coccyx. Triangle 2 is formed by lines tangent to the points of maximal inferomedial projection of the inner gluteal contours bilaterally. The ovoid representing the ideal gluteal shape is constructed with reference to these triangles: its major axis is directed toward the center of triangle 1, and its minor axis is perpendicular to triangle 2. The inferolateral extremity of the ovoid lies at the level of the IG line.

In the posterior view, rectangles and two ovoids may also be identified. An ovoid can be inscribed within the anatomical boundaries of the region delimited by the IS and IG lines (Fig. 2). Its orientation is defined such that the major axis is directed toward the center of the sacral triangle, whereas the minor axis is perpendicular to the inferior triangle. Consequently, the major axis of the ovoid is inclined at approximately 40° relative to the midline vertical axis.

## Discussion

In this brief report, we describe reference lines and geometric figures intended to provide insights into the aesthetics of the female body, with particular emphasis on the gluteal region. In our view, describing geometric parameters may contribute to a more systematic and standardized framework for characterization and preoperative planning.

Combining lateral and posterior reference lines allowed identification of multiple geometric configurations, including rectangles and ovoids, which may facilitate visualization of spatial relationships among gluteal structures.

Recently, interest has increased in generative artificial intelligence applications as tools for preoperative planning in plastic surgery. Among these, body contouring, liposculpture, and gluteoplasty—including the Brazilian butt lift—are particularly common. The development of such applications requires standardized guidelines for body proportions and target shapes that can serve as reference models. The geometric characteristics described here may support evaluation and preoperative planning for procedures involving the female gluteal region.

A key limitation of this work is the absence of clinical observations or pre- and postoperative measurements. The considerations presented reflect the authors’ aesthetic interpretation. Nonetheless, we hope these observations may serve as a basis for subsequent discussion. Future studies should assess whether, and to what extent, the proposed geometric figures correspond to clinical anatomy.

## Funding

None.

## Ethical approval

Not required.

## Declaration of competing interest

None declared.
